# Impact of Preanalytical Sample Preparation on the Recovery Rate of Cryopreserved Allogeneic Hematopoietic Stem Cells

**DOI:** 10.1111/ejh.70118

**Published:** 2026-01-19

**Authors:** Philipp Blüm, Christina Glas, Karen Bieback, Sabine Kayser, Harald Klüter, Patrick Wuchter

**Affiliations:** ^1^ Institute of Transfusion Medicine and Immunology, Medical Faculty Mannheim, Heidelberg University German Red Cross Blood Service Baden‐Württemberg ‐ Hessen Mannheim Germany

**Keywords:** cryopreserved allogeneic PBSCs, peripheral‐blood‐derived hematopoietic stem and progenitor cells, preanalytical sample preparation, quality control after cryopreservation, stem cell viability after cryopreservation

## Abstract

**Introduction:**

Cryopreservation of peripheral‐blood‐derived hematopoietic stem and progenitor cells (PBSCs) has been used for decades, primarily for autologous transplantation. Now, in the era of new cellular therapies, the cryopreservation of allogeneic PBSCs may also become relevant. While the defining quality‐control parameters are assessed before cryopreservation, in thawed cryopreserved samples the viability of total nucleated cells is often the only parameter analyzed. We therefore evaluated the recovery rates of total and viable CD45^+^ and CD34^+^ cells after cryopreservation.

**Methods:**

Thirty‐nine samples of allogeneic PBSC products were analyzed before and after cryopreservation. Post‐thaw viability was assessed using trypan blue exclusion. To investigate the influence of different preanalytical preparation methods, PBSCs were resuspended in phosphate‐buffered saline (PBS) or human serum albumin (HSA), and the cells suspended in HSA were tested without incubation or following incubation at 37°C for 1 h or 2 h. The number and viability of CD45^+^ and CD34^+^ cells were evaluated by flow cytometry and compared with pre‐thaw values.

**Results:**

After cryopreservation, we observed significant decreases in both total and viable CD45^+^ cell numbers. In contrast, the difference in total CD34^+^ cell numbers was rather small, whereas the median recovery rate of viable CD34^+^ cells after resuspension in PBS was 70.1%. Relevant differences between the preanalytical treatment groups could not be observed. The viability values as determined by trypan blue viability staining and flow cytometry were not correlated.

**Conclusion:**

Determination of the CD34^+^ cell recovery rate after thawing is feasible and may be used as an additional parameter for quality control for cryopreserved allogeneic PBSCs. However, the results of currently used viability assays vary considerably, making comparisons between different cell processing units and cryopreservation protocols difficult. Therefore, available protocols should be carefully evaluated based on “good‐manufacturing‐practice” (GMP) regulations before implementation.

## 1 Introduction

Hematopoietic stem and progenitor cells (HSPCs) have been used for the treatment of malignant hematological diseases for many decades [1, 2, 3, 4]. Allogeneic HSPC transplantation not only enables continuous healthy blood formation in recipients but also represents a strong immunotherapy directed against malignant cells (graft‐versus‐leukemia effect) [5, 6]. HSPCs can be collected from three different sources; accordingly, they are classified as bone‐marrow‐derived, cord‐blood‐derived, or mobilized peripheral‐blood‐derived HSPCs (PBSCs). Owing to several advantages, PBSCs are currently the most commonly collected and transplanted category of HSPCs, with many thousands of PBSC transplants performed worldwide each year [7, 8, 9].

Typically, fresh allogeneic PBSCs are stored at temperatures ranging from +2°C to +8°C for up to 72 h and must be transplanted within this time frame [10, 11, 12]. If there is a long distance between the PBSC collection center and the transplantation center, logistics can become quite challenging. To overcome the short shelf life of fresh PBSCs, cryopreservation of these cells, usually at temperatures ≤ −140°C, has been established as an option [13, 14], extending the shelf life to many years but increasing the complexity and cost of storage and shipment [15, 16, 17].

In the context of autologous use, cryopreservation represents the only option for PBSC storage, given the obligatory time gap between PBSC collection and high‐dose chemotherapy followed by autologous PBSC transplantation [13]. For allogeneic transplantation, fresh PBSCs are typically used, but during the COVID‐19 pandemic, cryopreservation was recommended by expert committees; fresh and cryopreserved PBSCs seem to be comparable in terms of product quality and patient outcomes [18, 19, 20, 21].

Unfortunately, cryopreservation protocols are generally not standardized between different collection sites and cell processing units [14]. In contrast to those that will be used fresh, PBSC suspensions that will be cryopreserved must be combined with a cryoprotective agent, most often dimethyl sulfoxide (DMSO), before the freezing process; the typical concentration of the cryoprotective agent is 5%–10% [13, 22, 23, 24]. This step is critical because DMSO protects PBSCs from cell death during freezing and facilitates cryopreserved storage [23].

For quality control, the CD45^+^ and CD34^+^ cell counts and cell viability are analyzed by flow cytometry directly after apheresis, before cell processing and cryopreservation. After cryopreservation, in many cell processing units, only the total nucleated cell (TNC) viability is measured, often by analyzing the cells via trypan blue exclusion microscopically, and a cutoff value of ≥ 70% viability is commonly applied [12]. After cryopreservation, CD45^+^ and CD34^+^ cell count and viability analysis by flow cytometry are usually not required by local regulations and authorities and are therefore often not performed [12].

Cell counts and viability analysis of thawed PBSC samples can be challenging [22, 25, 26, 27]. As a result of different DMSO concentrations and test protocols, the comparability of test results performed by different cell processing units is limited. In particular, there is a lack of defined preanalytical sample preparation recommendations that could support objective and reliable test results [25, 28]. In a brief report from the AABB‐ISCT joint working group cellular therapy product stability project team, Reich‐Slotky et al. [29] pointed out that there is no single assay available to predict the engraftment ability of cryopreserved stem cell products, which is generally accepted in the cell therapy community and by accreditation bodies (e.g., FACT, AABB, ICH) [29]. Especially, there is a lack of standardization for flow cytometry‐based post‐thaw viable target cell enumerations that need to be addressed [29]. Although generally prescriptive thresholds do not exist, cell processing units must validate their own manufacturing processes and must define product specificities and quality control parameters, which have to be checked regularly.

In this work, we investigated the influence of different preanalytical sample preparation protocols on the recovery rates of CD45^+^ and CD34^+^ cells in cryopreserved allogeneic PBSC samples. In the era of novel cell‐based immunotherapies, for example, CAR T‐cells, the transplantation of allogeneic PBSCs could evolve into new lines of treatment, creating new areas of interest in the cryopreservation of allogeneic PBSCs and the development of off‐the‐shelf PBSC products and PBSC biobanks. In this context, standardized protocols for quality control are essential and should be developed.

## 2 Methods

### 2.1 Sample Selection

Cryopreserved reference samples of 39 allogeneic PBSC apheresis products were analyzed. All apheresis sessions were performed at our institute (German Red Cross Blood Service Baden‐Württemberg—Hessen gGmbH, Institute of Transfusion Medicine and Immunology, Mannheim, Germany) between November 28, 2023, and December 28, 2023. All PBSC products had been ordered by and distributed to transplantation centers in accordance with international guidelines and recommendations as “fresh” products, without prior cryopreservation (storage/transport temperature: +4°C ± 2°C). To date, we have not been informed about any graft‐failures or product‐related adverse events.

Product reference samples were drawn and cryopreserved according to local quality control regulations and stored for retrospective analysis. All donors were informed ahead of PBSC apheresis and provided written informed consent for storage and analysis for research and quality control purposes. For cryopreservation, DMSO (CryoShure‐DMSO USP Grade, WAK–Chemie Medical GmbH, Steinbach, Germany) diluted in 5% human serum albumin (HSA) (albunorm 5%, Octapharma GmbH, Langenfeld, Germany) was added 1:1 to the apheresis sample for a final DMSO concentration of 7.5%. Two milliliters of each sample were subjected to controlled‐rate freezing using CoolCell LX (BioCision LLC, CA, USA) at −80°C and subsequently stored at temperatures of ≤ −150°C in the vapor phase of liquid nitrogen. Cryopreserved product reference samples had been stored for 13–15 months at the time of analysis.

All donors gave informed consent, and an Institutional Ethics Committee approved the analysis of the samples as well as the evaluation of the clinical data (Ref. 2025‐602).

### 2.2 Thawing and Viability Assessment With Trypan Blue

Thawing of the frozen samples was performed manually in a water bath at temperatures between 30°C and 40°C. To identify the phase transition from the solid phase to the liquid phase (melting point), the samples were visually monitored.

Directly after thawing, viability staining with trypan blue was conducted according to local standard procedures (Corning 100 mL Trypan Blue Solution, 0.4% (w/v) in PBS, pH 7.5 ± 0.5; Berlin, Germany). A total of 10 μL of each PBSC sample was mixed with 90 μL of trypan blue and incubated at room temperature for 1 min. For the cell viability analysis, we used a Neubauer hemocytometer and a light microscope to count the number of viable cells and the total number of cells.

### 2.3 Sample Preparation and Flow Cytometry Analysis

For flow cytometry analysis, four different preanalytical sample preparation protocols were compared: (1) resuspension in phosphate‐buffered saline (PBS) (DPBS 1×, Dulbecco's phosphate‐buffered saline, Gibco, Thermo Fisher Scientific, Dreieich, Germany); (2) resuspension in 5% human serum albumin (HSA 0 h); (3) resuspension in 5% HSA and incubation for 1 h at 37°C, 95% humidity and 5% CO_2_ (HSA 1 h); and (4) resuspension in 5% HSA and incubation for 2 h at 37°C, 95% humidity and 5% CO_2_ (HSA 2 h) (Heracell 240, Thermo Fisher Scientific, Dreieich, Germany).

**FIGURE 1:**
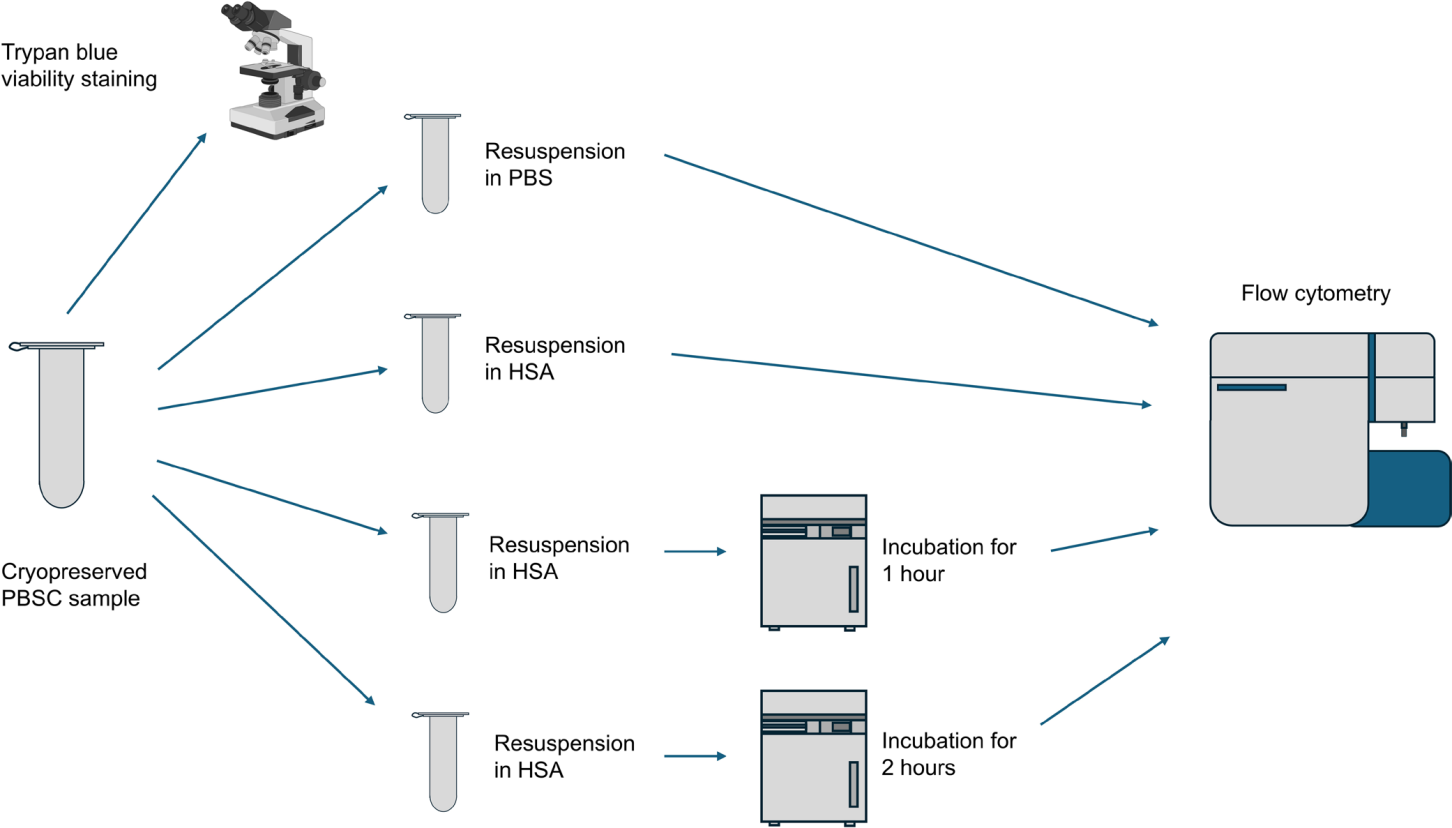
Experimental setup. Cryopreserved PBSC samples were manually thawed in a water bath. Directly after thawing, viability staining with trypan blue was conducted (standard method). In the experimental groups, the PBSC samples were resuspended in (1) PBS (1:5) or in HSA (1:5) followed by (2) no incubation; (3) HSA (1:5) incubation for 1 h at 37°C, 5% CO_2_ and 95% humidity; or (4) incubation for 2 h. Flow cytometry was used for CD45^+^ and CD34^+^ cell analysis.

In the PBS group, the samples were diluted 1:5 in PBS at room temperature (RT) and immediately analyzed by flow cytometry. In the HSA groups, 5% HSA was used instead of PBS to dilute the PBSCs at a ratio of 1:5. Additionally, flow cytometry was carried out directly after dilution with HSA (HSA 0 h); after dilution and 1 h of incubation at 37°C, 95% humidity and 5% CO_2_ (HSA 1 h); and after dilution and 2 h of incubation at 37°C, 95% humidity and 5% CO_2_ (HSA 2 h) (Figure 1).

For flow cytometry analysis, we used the single‐platform method according to the International Society of Hematotherapy and Graft Engineering (ISHAGE) guidelines [30, 31]. A 100 μL volume of each PBSC sample was mixed with 40 μL of the “BD Stem Cell Enumeration Kit” reagent in a “BD Trucount tube” (BD Biosciences, Heidelberg, Germany). Viability analysis was performed with 7‐aminoactinomycin D (7‐AAD), which is included in the “BD Stem Cell Enumeration Kit.” Anti‐CD45 was used for the identification of white blood cells (WBCs), and anti‐CD34 was used for the enumeration of HSPCs (also part of the BD Stem Cell Enumeration Kit). After incubation in 2000 μL of lysis buffer, flow cytometry analysis was performed with a BD FACSLyric flow cytometer and BD FACSuite v1.5 software (BD Biosciences, Heidelberg, Germany). According to the ISHAGE protocol, the following gating strategy and stopping criteria were used: ≥ 75 000 CD45^+^ cells, ≥ 100 CD34^+^ cells, and ≥ 1000 BD Trucount beads. This flow cytometry setting was used for all samples, and the results were compared to those of fresh samples.

The target cell concentration and recovery rate were calculated as follows:

$$\begin{array}{c} \text{Target cell concentration}\;\left(\frac{n}{\mathrm{\mu L}}\right)\\ =\frac{\text{target cell number}\;\left(n\right)\times \text{Trucount beads}\;\mathrm{per}\;\text{tube}\times \text{dilution factor}\;}{\text{bead number}\times 100\mathrm{\mu L}\;\text{sample volume}} \end{array}$$

$$\begin{array}{c} \text{Recovery rate}\;\left(\%\right)\\ =\frac{\text{target cell concentration after cryopreservation}}{\text{target cell concentration before cryopreservation}}\times 100 \end{array}$$

### 2.4 Statistical Analysis

For statistical analyses, GraphPad Prism software version 9.3.1 (GraphPad Software LLC, Boston, MA, USA) was used. To compare the means of two normally distributed groups, a paired t test was used, with a significance level of 0.05. To compare the means of more than two groups, we used one‐way ANOVA with a significance level of 0.05. For the linear correlation between trypan blue viability staining and viability assessment with 7‐AAD, we used the Pearson correlation coefficient.

## 3 Results

### 3.1 Donor and Apheresis Characteristics

Thirty‐nine allogeneic apheresis sessions and the retained cryopreserved product samples from those sessions were analyzed (Table 1). Twenty‐nine (74.4%) donors were male, and 10 (25.6%) were female. The median donor age was 27 years (range: 18–43 years), the median body weight was 83 kg (range: 62–107 kg), and the median body surface area was 2.05 m^2^ (range: 1.68–2.40 m^2^).

**TABLE 1:** Donor and apheresis characteristics.

Donor characteristics	
Total number of apheresis sessions analyzed, *n*	39
Male, no. (%)	29 (74.4)
Female, no. (%)	10 (25.6)
Age in years, median (range)	27 (18–43)
Body weight in kg, median (range)	83 (62–107)
Body surface area in m^2^, median (range)	2.05 (1.68–2.40)
Blood count ahead of apheresis	
Leukocytes × 10^9^/L, median (range)	41.3 (16.9–98.7)
Hematocrit in %, median (range)	42.0 (34.9–47.8)
Thrombocytes × 10^9^/L, median (range)	193 (96–380)
Viable CD34^+^ cells in % of WBC, median (range)	0.13 (0.04–0.42)
Viable CD34^+^ cells per μL, median (range)	50.5 (12.9–220.8)
Apheresis/product characteristics	
Processed blood volume in l, median (range)	11.4 (4.8–19.3)
Product volume in ml, median (range)	324 (122–369)
Leukocytes × 10^9^/L, median (range)	196 (120–253)
Hematocrit in %, median (range)	2.2 (0.8–3.0)
Thrombocytes × 10^9^/L, median (range)	1843 (565–2883)
Viable CD34^+^ cells in % of WBC, median (range)	0.75 (0.27–2.21)
Viable CD34^+^ cells per μL, median (range)	1469 (370–4176)
Total number of nucleated cells × 10^9^, median (range)	60.7 (19.6–89.1)
Total number of CD34^+^ cells × 10^6^, median (range)	452 (107–981)
Viability CD45^+^ cells in %, median (range)	100 (99–100)
Viability CD34^+^ cells in %, median (range)	99 (92–100)

The blood count ahead of apheresis showed typical values after stem cell mobilization with granulocyte colony‐stimulating factor (G‐CSF or filgrastim): median leukocyte count—41.3 × 10^9^/L (range: 16.9–98.7 × 10^9^/L); median hematocrit level—42.0% (range: 34.9%–47.8%); and median platelet count—193 × 10^9^/L (range: 96–380 × 10^9^/L). In the peripheral blood before stem cell apheresis, the median frequency of viable CD34^+^ cells was 0.13% (range: 0.04%–0.42%) of nucleated cells, or 50.5/μL (range: 12.9–220.8/μL).

During apheresis, a median of 11.4 L (range: 4.8–19.3 L) of donor blood was processed, and a median product volume of 324 mL (range: 122–369 mL) was collected.

The median concentration of nucleated cells in the product was 196 × 10^9^/L (range: 120–253 × 10^9^/L), the median hematocrit value was 2.2% (range: 0.8%–3.0%), and the median platelet concentration was 1843 × 10^9^/L (range: 565–2883 × 10^9^/L).

The median proportion of viable CD34^+^ cells in the product was 0.75% (range: 0.27%–2.21%) of nucleated cells, and the median CD34^+^ cell concentration in the product was 1469/μL (range: 370–4176/μL). The median total number of nucleated cells in the product was 60.7 × 10^9^ (range: 19.6–89.1 × 10^9^), and the median total number of CD34^+^ cells in the product was 452 × 10^6^ (range: 107–981 × 10^6^).

### 3.2 CD45^+^ and CD34^+^ Cell Concentration and Viability at the End of Apheresis

The median concentration of total CD45^+^ cells in the apheresis product at the end of apheresis was 195.3 × 10^9^/L (range: 105.8–239.6 × 10^9^/L), the median concentration of viable CD45^+^ cells was 194.6 × 10^9^/L (range: 105.5–238.5 × 10^9^/L), the median concentration of total CD34^+^ cells was 1.48 × 10^9^/L (range: 0.38–4.19 × 10^9^/L), and the median concentration of viable CD34^+^ cells was 1.47 × 10^9^/L (range: 0.37–4.18 × 10^9^/L). As measured by flow cytometry, the median viability of the CD45^+^ cells was 100% (range: 99%–100%), and the median viability of the CD34^+^ cells was 99% (range: 92%–100%) (Table 2).

**TABLE 2:** Overview of cell viabilities.

Fresh product before cryopreservation, *n* = 39						
Condition	Parameter	Median	Min	Max	Mean	SD
Apheresis product	CD45^+^ viability, %	100	99	100	99.9	0.4
	CD34^+^ viability, %	99	92	100	99.0	1.5

Thawed cryopreserved samples, *n* = 39						
Condition	Parameter	Median	Min	Max	Mean	SD
PBS	CD45^+^ viability, %	68	51	81	67.5	6.6
	CD34^+^ viability, %	77	53	87	74.3	7.9
	Viable CD45^+^ cell recovery rate, %	56	40	80	58.8	10.1
	Viable CD34^+^ cell recovery rate, %	70	44	106	71.4	13.5
HSA 0 h	CD45^+^ viability, %	67	49	79	65.5	6.3
	CD34^+^ viability, %	74	54	85	72.5	7.5
	Viable CD45^+^ cell recovery rate, %	60	32	93	61.3	11.4
	Viable CD34^+^ cell recovery rate, %	77	47	118	76.5	13.8
HSA 1 h	CD45^+^ viability, %	69	59	78	68.6	4.6
	CD34^+^ viability, %	79	54	86	76.3	7.8
	Viable CD45^+^ cell recovery rate, %	59	34	81	57.8	10.9
	Viable CD34^+^ cell recovery rate, %	83	57	111	83.6	12.4
HSA 2 h	CD45+ viability, %	67	58	76	66.9	4.5
	CD34+ viability, %	70	34	82	68.3	11.0
	Viable CD45^+^ cell recovery rate, %	56	25	76	54.8	11.6
	Viable CD34^+^ cell recovery rate, %	81	52	100	79.7	12.1

Abbreviation: SD, standard deviation.

### 3.3 Influence of Different Treatment Conditions on the CD45^+^ and CD34^+^ Cell Concentration and Viability After Thawing of Frozen PBSC Samples

**FIGURE 2:**
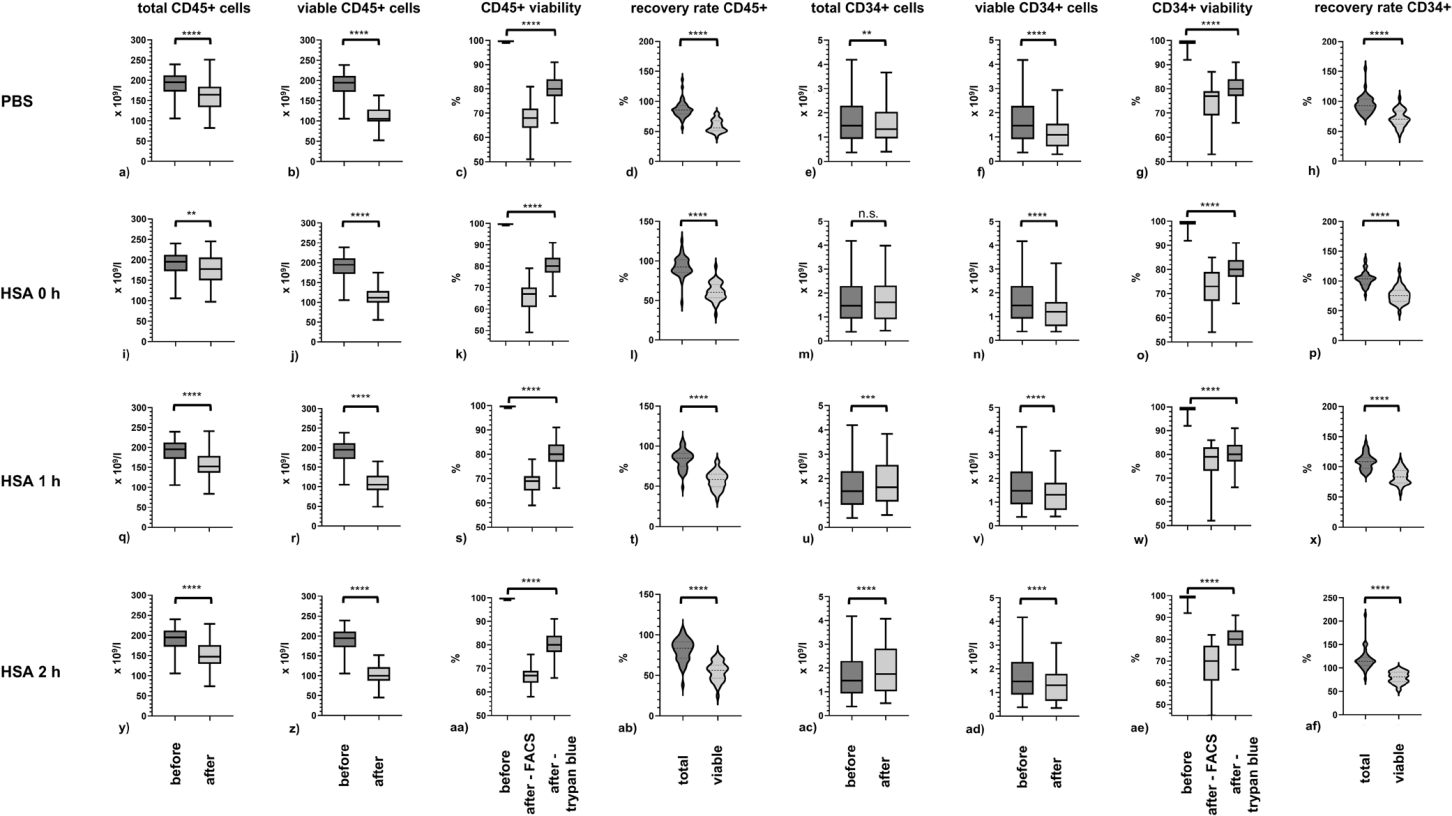
CD45^+^ and CD34^+^ cells after thawing and resuspension in PBS and in HSA. (a) Total CD45^+^ cells before and after cryopreservation and resuspension in PBS; (b) viable CD45^+^ cells before and after cryopreservation and resuspension in PBS; (c) CD45^+^ viability before and after cryopreservation and resuspension in PBS as measured by flow cytometry and trypan blue staining; (d) recovery rates of total and viable CD45^+^ cells after resuspension in PBS; (e) total CD34^+^ cells before and after cryopreservation and resuspension in PBS; (f) viable CD34^+^ cells before and after cryopreservation and resuspension in PBS; (g) CD34^+^ viability before cryopreservation and after cryopreservation and resuspension in PBS as measured by flow cytometry and trypan blue staining; (h) recovery rates of total and viable CD34^+^ cells after resuspension in PBS; (i) total CD45^+^ cells before and after cryopreservation and resuspension in HSA; (j) viable CD45^+^ cells before and after cryopreservation and resuspension in HSA; (k) CD45^+^ viability before and after cryopreservation and resuspension in HSA as measured by flow cytometry and trypan blue staining; (l) recovery rates of total and viable CD45^+^ cells after resuspension in HSA; (m) total CD34^+^ cells before and after cryopreservation and resuspension in HSA; (n) viable CD34^+^ cells before and after cryopreservation and resuspension in HSA; (o) CD34^+^ viability before and after cryopreservation and resuspension in HSA as measured by flow cytometry and trypan blue staining; (p) recovery rates of total and viable CD34^+^ cells after resuspension in HSA; (q) total CD45^+^ cells before and after cryopreservation, resuspension in HSA and incubation for 1 h; (r) viable CD45^+^ cells before and after cryopreservation, resuspension in HSA and incubation for 1 h; (s) CD45^+^ viability before and after cryopreservation, resuspension in HSA and incubation for 1 h as measured by flow cytometry and trypan blue staining; (t) recovery rates of total and viable CD45^+^ cells after resuspension in HSA and incubation for 1 h; (u) total CD34^+^ cells before and after cryopreservation, resuspension in HSA and incubation for 1 h; (v) viable CD34^+^ cells before and after cryopreservation, resuspension in HSA and incubation for 1 h; (w) CD34^+^ viability before cryopreservation and after cryopreservation, resuspension in HSA and incubation for 1 h as measured by flow cytometry and trypan blue staining; (x) recovery rates of total and viable CD34^+^ cells after resuspension in HSA and incubation for 1 h; (y) total CD45^+^ cells before and after cryopreservation, resuspension in HSA and incubation for 2 h; (z) viable CD45^+^ cells before and after cryopreservation, resuspension in HSA and incubation for 2 h; (aa) CD45^+^ viability before and after cryopreservation, resuspension in HSA and incubation for 2 h as measured by flow cytometry and trypan blue staining; (ab) recovery rates of total and viable CD45^+^ cells after resuspension in HSA and incubation for 2 h; (ac) total CD34^+^ cells before and after cryopreservation, resuspension in HSA and incubation for 2 h; (ad) viable CD34^+^ cells before and after cryopreservation, resuspension in HSA and incubation for 2 h; (ae) CD34^+^ viability before and after cryopreservation and resuspension in HSA and incubation for 2 h as measured by flow cytometry and trypan blue staining; (af) recovery rates of total and viable CD34^+^ cells after resuspension in HSA and incubation for 2 h. *n* = 39. (a–c, e–g, i–k, m–o, q–s, u–w, y–aa, ac–ae) box‐and‐whisker plots; (d, h, l, p, t, x, ab, af) violin plots; (a, b, d–j, l–n, p–r, t–z, ab–ad, af) paired *t* test: N.s.—not significant, **p < 0.01, ***p < 0.001, ****p < 0.0001; (c, g, k, o, s, w, aa, ae) one‐way ANOVA: ****p < 0.0001.

Flow cytometric analysis was performed after thawing and then resuspending the frozen PBSC samples in PBS, in HSA without incubation, in HSA and incubation for 1 h, and for 2 h at 37°C, 5% CO_2_ and 95% humidity (Figure 2).

The median concentrations of total CD45^+^ cells were 164.2 × 10^9^/L (range: 82.6–251.1 × 10^9^/L), 176.8 × 10^9^/L (range: 97.5–245.3 × 10^9^/L), 152.1 × 10^9^/L (range: 83.8–241.0 × 10^9^/L), and 147.3 × 10^9^/L (range: 73.5–228.5 × 10^9^/L), respectively. The median concentrations of viable CD45^+^ cells for each condition were 105.3 × 10^9^/L (range: 52.1–163.2 × 10^9^/L), 111.4 × 10^9^/L (range: 55.6–174.8 × 10^9^/L), 105.8 × 10^9^/L (range: 49.2–165.3 × 10^9^/L), and 100.0 × 10^9^/L (range: 45.1–152.0 × 10^9^/L).

The median concentrations of total CD34^+^ cells were 1.3 × 10^9^/L (range: 0.4–3.7 × 10^9^/L), 1.6 × 10^9^/L (range: 0.4–4.0 × 10^9^/L), 1.6 × 10^9^/L (range: 0.5–3.8 × 10^9^/L), 1.8 × 10^9^/L (range: 0.5–4.1 × 10^9^/L) for samples resuspended in PBS, in HSA without incubation, in HSA and incubation for 1 h, and 2 h. The median concentrations of viable CD34+ cells were 1.1 × 10^9^/L (range: 0.3–2.9 × 10^9^/L), 1.2 × 10^9^/L (range: 0.4–3.3 × 10^9^/L), 1.3 × 10^9^/L (range: 0.4–3.2 × 10^9^/L), and 1.3 × 10^9^/L (range: 0.3–3.1 × 10^9^/L), respectively.

These results represent a median total CD45^+^ cell recovery rate of 85.9% (range: 56.3%–136.4%), 92.2% (range: 47.2%–125.9%), 85.2% (range: 48.8%–107.5%), and 83.5% (range: 38.2%–106.0%). The median recovery rates of viable CD45^+^ cells were 56.0% (range: 40.3%–80.3%), 60.3% (range: 31.9%–93.2%), 58.7% (range: 33.9%–81.4%), and 56.1% (range: 24.7%–75.8%).

The median recovery rates of total CD34^+^ cells were 92.8% (range: 73.6%–154.9%), 104.0% (range: 76.5%–137.2%), 108.9% (range: 87.3%–140.6%), and 114.6% (range: 77.3%–213.6%). The median recovery rates of viable CD34^+^ cells were 70.1% (range: 43.8%–105.7%), 76.5% (range: 46.9%–118.2%), 83.3% (range: 57.0%–111.1%), and 81.1% (range: 52.3%–100.0%), respectively.

### 3.4 Comparison of Cell Viability Measured by Flow Cytometry and Trypan Blue

The median cell viability after cryopreservation as measured by trypan blue viability staining was 80% (range: 66%–91%). The median CD45^+^ cell viability values after cryopreservation as measured by flow cytometry after resuspension in PBS, after resuspension in HSA, after resuspension in HSA and incubation for 1 h, and after resuspension in HSA and incubation for 2 h were 68% (range: 51%–81%), 67% (range: 49%–79%), 69% (range: 59%–78%), and 67% (range: 58%–76%), respectively. The median CD34^+^ cell viability values as measured by flow cytometry after resuspension in PBS, after resuspension in HSA, after resuspension in HSA and incubation for 1 h, and after resuspension in HSA and incubation for 2 h were 77% (range: 53%–87%), 74% (range: 54%–85%), 79% (range: 54%–86%), and 70% (range: 34%–82%), respectively.

The viability values measured by trypan blue for total nucleated cells (TNCs) and the viability values evaluated for CD45^+^ and CD34^+^ cells by flow cytometry for each condition did not correlate. The Pearson correlation coefficients for trypan blue and flow cytometry for CD45^+^ cells were *r* = 0.25 (95% confidence interval (CI) −0.07 to 0.53) for PBS, *r* = 0.19 (95% CI −0.13 to 0.48) for HSA, *r* = 0.27 (95% CI −0.05 to 0.54) for HSA 1 h, and *r* = 0.31 (95% CI −0.01 to 0.57) for HSA 2 h. The Pearson correlation coefficients for trypan blue and flow cytometry for CD34^+^ cells were *r* = −0.07 (95% CI −0.38 to 0.24) for PBS, *r* = 0.11 (95% CI −0.22 to 0.41) for HSA, *r* = −0.04 (95% CI −0.35 to 0.28) for HSA 1 h, and *r* = −0.22 (95% CI −0.50 to 0.11) for HSA 2 h.

### 3.5 Comparison of Total and Viable CD45 ^+^ and CD34 ^+^ Cell Counts Between Different Conditions

**FIGURE 3:**
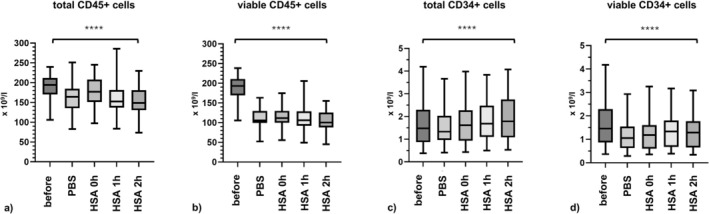
Total and viable CD45^+^ and CD34^+^ cells under different treatment conditions. Flow cytometry analysis of (a) total CD45^+^ cells, (b) viable CD45^+^ cells, (c) total CD34^+^ cells, and (d) viable CD34^+^ cells. *n* = 39. Box‐and‐whisker plots for before cryopreservation (before), after cryopreservation and resuspension in PBS (PBS), after resuspension in HSA without incubation (HSA 0 h), after resuspension in HSA and incubation for 1 h (HSA 1 h), and after resuspension in HSA and incubation for 2 h (HSA 2 h). One‐way ANOVA: ****p < 0.0001.

Although there were statistically significant differences in the numbers of total CD45^+^, viable CD45^+^, total CD34^+^, and viable CD34^+^ cells between the different treatment groups and the fresh samples (one‐way ANOVA for each group, *n* = 39, *p* < 0.0001), the absolute differences between the means were minimal (Figure 3).

The mean concentrations of total CD45^+^ cells after thawing and resuspension in PBS, in HSA without incubation, in HSA and incubation for 1 h, and 2 h, were 163.0 × 10^9^/L, 173.1 × 10^9^/L, 155.9 × 10^9^/L, and 151.4 × 10^9^/L. All values were significantly lower than the mean concentration of total CD45^+^ cells after apheresis, at 187.7 × 10^9^/L (paired *t* test, *n* = 39, HSA without incubation *p* < 0.01, PBS, HSA 1 h, HSA 2 h *p* < 0.0001).

The mean concentrations of viable CD45^+^ cells measured after thawing for each sample treatment condition were 109.6 × 10^9^/L, 113.7 × 10^9^/L, 107.4 × 10^9^/L, and 101.7 × 10^9^/L. These results were significantly lower than the mean concentration of viable CD45^+^ cells after apheresis, that is, 187.0 × 10^9^/L (paired t test, *n* = 39, *p* < 0.0001).

The mean concentrations of total CD34^+^ cells for each condition were 1.6 × 10^9^/L, 1.7 × 10^9^/L, 1.8 × 10^9^/L, and 1.9 × 10^9^/L. The mean concentration of total CD34+ cells resuspended in PBS was significantly lower than the mean concentration of total CD34^+^ cells after apheresis, that is, 1.7 × 10^9^/L (paired *t* test, *n* = 39, *p* = 0.0027). In contrast, the value of samples resuspended in HSA without incubation was not significantly different from the mean concentration of total CD34^+^ cells after apheresis (paired *t* test, *n* = 39, *p* = 0.12). Interestingly, the mean concentration of total samples resuspended in HSA and incubation for 1 h, as well as for 2 h were significantly greater than the mean concentration of total CD34^+^ cells after apheresis (paired t test, *n* = 39, HSA 1 h *p* < 0.001, HSA 2 h *p* < 0.0001). Of note, although statistically significant, the absolute differences between these values were only small.

Finally, the mean concentrations of viable CD34^+^ cells after thawing were 1.2 × 10^9^/L, 1.3 × 10^9^/L, 1.4 × 10^9^/L, and 1.4 × 10^9^/L, respectively. All values were significantly lower than the mean concentration of viable CD34^+^ cells after apheresis, that is, 1.7 × 10^9^/L (paired *t* test, *n* = 39, < 0.0001).

## 4 Discussion

The cryopreservation of PBSCs for long‐term storage is a well‐established procedure and has been used for many decades [13, 21, 23]. For quality‐control purposes, cell enumeration and viability assessments are typically performed before cryopreservation. The composition and specifications of the final product are derived mainly from these values. Cell counting and cell viability analysis following cryopreservation are known to be difficult [25, 27, 32, 33]. This difficulty is related to the cryopreservation and thawing processes, both of which are relevant stressors that are responsible for cell death and cell loss. Many cell processing units use viability staining with trypan blue and microscopy, a method characterized by high interobserver variability [25, 27, 29, 33]. Flow cytometry is an alternative, but viability data obtained by 7‐AAD staining are typically much lower than those obtained by trypan blue staining [29]. We aimed to assess the impact of preanalytical methods on the recovery and viability of PBSCs after cryopreservation.

### 4.1 Decreased CD45 ^+^ Cell Count and Viability After Cryopreservation

As expected, we observed significant decreases in the numbers of total CD45^+^ cells and viable CD45^+^ cells after cryopreservation. This decrease was consistently detectable for each treatment group: resuspension in PBS, resuspension in HSA without incubation, resuspension in HSA followed by 1 h of incubation, and resuspension in HSA followed by 2 h of incubation. This effect seems to be driven mainly by the death of CD34‐negative cells, since the recovery rates for total and viable CD34^+^ cells were markedly higher. Cai et al. [25] reported that granulocytes and T cells are the cells most vulnerable to loss with cryopreservation.

The results of the viability analysis by flow cytometry (7‐AAD) were consistently lower and showed greater variance than the viability values determined by trypan blue staining. The absolute values of the total and viable CD45^+^ cell concentrations of the different treatment groups were comparable. The effect of preanalytical sample preparation of cryopreserved PBSCs appears to be negligible, as all measurable cell death in all treatment groups had already occurred before sample preparation.

### 4.2 Similar CD34 ^+^ Cell Counts but Decreased Viability After Cryopreservation

In contrast to total CD45^+^ cells, the subgroup of total CD34^+^ cells after cryopreservation presented a median recovery rate of 90%, with some values > 100%. This phenomenon was observed in all the treatment groups. Interestingly, the total CD34^+^ cell concentrations before and after cryopreservation for thawed PBSC samples resuspended in HSA without incubation were not significantly different. The absolute numbers of total CD34^+^ cells and viable CD34^+^ cells before and after cryopreservation were within a narrow range for all preanalytical sample preparation protocols. In line with observations for all CD45^+^ cells, the reduced number of viable CD34+ cells was most likely the result of cryopreservation effects and, to a lesser extent, was related to post‐thaw sample preparation methods.

### 4.3 Cell Viability Values Assessed by Trypan Blue Staining and Flow Cytometry Were Not Correlated

The cell viability values assessed by trypan blue staining and flow cytometry were not correlated. In all the treatment groups, the viability of CD45^+^ and CD34^+^ cells determined by flow cytometry was significantly lower than that determined by trypan blue. When the values from the two methods were compared directly, the viability values did not show a linear correlation, indicating that a comparison of the results of trypan blue viability staining and flow cytometry was not possible.

Importantly, trypan blue staining can only evaluate the viability of total nucleated cells (TNCs), whereas flow cytometry makes it possible to discriminate the viability of CD45^+^ and CD34^+^ cells. Furthermore, cells with positive 7‐AAD staining will also include early and late apoptotic cells along with necrotic cells, whereas cells with positive trypan blue staining will include only necrotic cells fully permeable to the dye. This explains the discrepancy between the methods in terms of viability.

### 4.4 Cell Viability of Cryopreserved Hematopoietic Stem Cells: Advantages and Limitations of Flow Cytometry

The concept of “hematopoietic stem cell viability” and the optimal method for its assessment have been subjects of scientific debate for decades [29]. Flow cytometry offers notable advantages, including a rapid turnaround time and the potential for standardization. Considerable efforts have been made to harmonize flow cytometric test results across different laboratories. The protocol of the “International Society of Hematotherapy and Graft Engineering (ISHAGE)” is widely recognized by accreditation bodies, for example, the Foundation for the Accreditation of Cellular Therapy/Joint Assurance Committee—ISCT and EBMT (FACT/JACIE) [30, 31, 34]. According to the 8th edition of the FACT/JACIE “hematopoietic cellular therapy accreditation manual,” viability is defined as “living cells as defined by dye exclusion, flow cytometry, or progenitor cell culture”. Section D8.1.3 specifies that “established, appropriate, and validated assays” should include total nucleated cell (TNC) count and viability, and CD34 enumeration and viability assays [34]. However, these standards do not define a specific test method, instead requiring each stem cell processing facility to validate its own appropriate test procedures.

The AABB‐ISCT Joint Working Group Stability Project Team categorizes HSPC stability assays in three groups: (1) quantification, (2) viability, and (3) function [29]. For quantification and viability, the ISHAGE protocol is considered highly suitable and has been largely standardized across laboratories [29, 30, 31]. In addition to the ISHAGE protocol, which is regarded as the gold standard flow cytometric product quality assessment, other flow cytometric methods are available for cell counting and viability analysis [35, 36]. For the evaluation of cell viability, besides 7‐AAD staining, additional markers such as aldehyde dehydrogenase (ALDH) activity [37] and annexin V staining [38, 39] have been investigated and correlated with colony forming unit (CFU) assays. Nevertheless, none of these approaches directly measure HSPC potency in a functional manner. Consequently, CFU assays remain valuable for evaluating the clonogenic potential of HSPCs [29], but have a long turnaround time, lack full standardization across laboratories [29, 40], and require clear definitions for appropriate read‐outs and quantitative evaluation of proliferative potential [41]. Currently, CFU assays between different cell processing units may vary considerably, and internationally accepted standards and guidelines are lacking [40]. In conclusion, while flow cytometric assays cannot directly assess HSPC potency, they provide significant advantages over CFU assays, making them well suited for quality control analysis of HSPC products.

### 4.5 Relevance of the Quality‐Control Analysis of Cryopreserved PBSC Samples

The fundamental problem of quality‐control analysis of thawed PBSC samples is that the most relevant product specifications are measured directly after apheresis from fresh product samples. Thresholds for minimal CD34^+^ cell counts and cell viability per graft, for example, ≥ 2 × 10^6^/kg body weight for autologous transplantation and ≥ 4 × 10^6^/kg body weight for allogeneic transplantation, are related to fresh product samples. To date, it remains unclear whether different protocols used by different cell processing units, for example, different DMSO concentrations and different cryopreservation protocols, have a relevant impact on product quality, given that the available quality control methods used after cryopreservation, primarily viability analysis by trypan blue or flow cytometry, are not correlated. In particular, the enumeration of total nucleated cells, CD45^+^ cells and/or CD34^+^ cells after cryopreservation is often not required and is therefore not routinely performed [29]. This makes comparisons of product quality between different cell processing units, cell processing protocols, and, ultimately, different manufacturers difficult or even impossible.

Nonetheless, in this study, we demonstrated a median viable CD34^+^ cell recovery rate of 70.1% (range: 43.8%–105.7%, PBS group), as measured by flow cytometry. The rather high variance of 43.8%–105.7% is remarkable and difficult to explain. Since all viability measurements have been performed under standardized conditions, with the same flow cytometric reagents and at the same flow cytometer, and the cryopreservation of the sample was performed using the identical manufacturing protocol, these differences may be related to donor‐specific effects. One should keep in mind that the flow cytometric viability assessment using 7‐AAD has its limitations, especially in regard to predicting stem cell engraftment and potency [29]. We consider these values to be plausible, and in comparison with previous studies with autologous HSPC products, their variance is actually much narrower [26]. This may be due in part to the fact that we analyzed PBSCs from healthy allogeneic donors, processed under optimal conditions in terms of the use of identical freezing procedures and cryopreserved for only a relatively short duration (less than 16 months). In summary, the re‐enumeration of allogeneic CD34^+^ cells after cryostorage seems feasible with the tested preanalytical thawing conditions.

## 5 Conclusion

Evaluating the quality‐control parameters of cryopreserved PBSCs is challenging, limiting the comparability of test results between different cell processing units and making the definition of universal thresholds difficult.

We observed that the cell viability of cryopreserved PBSC samples was, as expected, lower than the viability of fresh product samples at the end of apheresis. Post‐thaw PBSC sample preparation protocols have only a limited effect on the cell concentration and viability, and the absolute differences between the treatment groups were small. The cell viability values evaluated by trypan blue viability staining and flow cytometry (7‐AAD) were not correlated.

The re‐enumeration of allogeneic CD34^+^ cells after cryostorage may be a useful tool in the future, but standardized protocols must be developed, and reliable quality‐control parameters should be defined and implemented in international guidelines and recommendations.

## Author Contributions

P.B. analyzed the data and wrote the manuscript. C.G. performed the experiments. S.K. supervised the leukapheresis sessions and revised the manuscript. K.B. and H.K. provided conceptual advice and revised the manuscript. P.W. planned and designed the experiments, provided conceptual advice, and revised the manuscript.

## Funding

This study was supported by research funding from the German Red Cross Blood Service Baden‐Württemberg—Hessen.

## Ethics Statement

An Institutional Ethics Committee approved the analysis of the samples as well as the evaluation of the clinical data (Ref. 2025‐602).

## Consent

All donors gave informed consent.

## Conflicts of Interest

The authors declare no conflicts of interest.

## Data Availability

The data that support the findings of this study are available on request from the corresponding author. The data are not publicly available due to privacy or ethical restrictions.
